# Whole-body PET/CT reveals systemic dissemination in spinal tuberculosis: findings from the Spinal TB X cohort

**DOI:** 10.1007/s15010-026-02750-w

**Published:** 2026-03-03

**Authors:** Julian Scherer, Sandra L. Mukasa, Karen Wolmarans, Fareda Jakoet-Bassier, Ashleigh Taylor, Reto Guler, Tessa Kotze, Taeksun Song, Robert Dunn, Maritz Laubscher, Hans-Christoph Pape, Michael Held, Friedrich Thienemann

**Affiliations:** 1 General Medicine and Global Health Research Unit, Department of Medicine, Faculty of Health Science, University of Cape Town, 4th Floor, Chris Barnard Building, 3 Anzio Road, Cape Town, 7925 South Africa; 2 Orthopaedic Research Unit, Division of Orthopaedic Surgery, Department of Surgery, Faculty of Health Science, University of Cape Town, Cape Town, South Africa; 3 Institute of Infectious Diseases and Molecular Medicine and Division of Immunology, Department of Pathology, Faculty of Health Science, University of Cape Town, Cape Town, South Africa; 4 International Centre for Genetic Engineering and Biotechnology, Cape Town Component, Cape Town, South Africa; 5 Cape Universities Body Imaging Centre, Faculty of Health Science, University of Cape Town, Cape Town, South Africa; 6 Institute of Infectious Diseases and Molecular Medicine and Division of Medical Microbiology, Department of Pathology, Faculty of Health Sciences, University of Cape Town, Cape Town, South Africa; 7 Department of Traumatology, University Hospital Zurich, University of Zurich, Zurich, Switzerland; 8 Department of Internal Medicine, University Hospital Zurich, University of Zurich, Zurich, Switzerland

**Keywords:** Tuberculosis, Spondylodiscitis, Spinal, Extrapulmonary, PET/CT

## Abstract

**Introduction:**

Spinal tuberculosis (STB) is a severe form of extrapulmonary tuberculosis associated with diagnostic delay and substantial morbidity. The extent of disease—isolated versus disseminated STB—remains poorly defined. We applied whole-body 18F-FDG-PET/CT to characterise the clinical and radiographic phenotype of STB.

**Material and methods:**

The Spinal TB X cohort (NCT05610098) is a prospective study of HIV-infected and uninfected patients with suspected STB in Cape Town, South Africa. We analysed the first ten patients with microbiologically confirmed STB. Demographic, clinical, microbiological, histological, and PET/CT data were collected. Isolated STB was defined as spinal involvement only, while disseminated STB was defined as additional organ involvement confirmed by microbiology and/or PET/CT. The study was approved by the Human Research Ethics Committee of the University of Cape Town (HREC 243/2022). Written informed consent was obtained from all participants.

**Results:**

Ten patients (median age 44 years [IQR 26], 80% male, 30% HIV-positive) were included. A total of 14 spinal lesions involving 44 vertebrae were detected; psoas abscesses were present in six patients. Mean SUVmax and SUVmean of spinal lesions were 15.2 (SD 4.6) and 6.9 (SD 1.5), respectively; mean total lesion glycolysis (TLG) was 363.2 SUVbw*mL (SD 341.9). Based on microbiology alone, 40% had disseminated STB, combining microbiology with PET/CT findings, 60% had disseminated disease.

**Conclusion:**

Whole-body PET/CT revealed a high frequency of disseminated disease, underscoring the systemic nature of TB even when clinical presentation is spinal. Isolated STB may represent a distinct phenotype. PET/CT shows promise for assessing disease burden and warrants further evaluation.

## Introduction

Each year, more than 10 million individuals develop tuberculosis (TB), caused by *Mycobacterium tuberculosis* (Mtb). Spinal tuberculosis (STB), a severe form of extrapulmonary TB (EPTB) leading to spondylitis or spondylodiscitis, typically presents with localized back pain, systemic debility, spinal deformity with or without instability, neurological impairment, and constitutional symptoms. The interval between symptom onset and definitive diagnosis may extend over several years. [1–3] Skeletal TB accounts for up to 20% of all EPTB cases, with spinal involvement occurring in approximately half of these. Immunosuppressed individuals, particularly people living with HIV, are at increased risk of developing EPTB and its complications. [4, 5] Diagnosis of STB relies on clinical assessment, spinal imaging, and microbiological confirmation from biopsy specimens. However, diagnosis is often delayed due to the nonspecific nature of symptoms and the limited sensitivity of available diagnostic methods. [6] Magnetic resonance imaging (MRI) is the gold standard for evaluating suspected STB, with a reported sensitivity of 94% and specificity of 93% for detecting spondylodiscitis. [7] MRI enables accurate assessment of spinal cord compression, intrinsic cord changes, vertebral body involvement, disc destruction, and psoas abscess formation. [8] Whole-spine MRI is recommended whenever feasible, as non-contiguous lesions are observed in up to 20% of patients [9].

Little is known about the full extent of disease in STB. Both isolated STB, confined to the vertebrae and adjacent anatomical structures, and disseminated forms with involvement of other organs, such as the lungs, lymph nodes, or the urogenital tract, have been described. [10–12].

Whether these two forms represent distinct disease entities or variations along a continuum remains unclear. The diagnosis of disseminated STB requires whole-body imaging and/or microbiological confirmation of *Mtb* from additional sites of infection [13].

2-deoxy-2-[18F]-fluoroglucose positron emission tomography/computed tomography (PET/CT) has emerged as a promising modality for detecting sites of infection and monitoring treatment response in TB. [14] FDG accumulates in tissues with increased glucose metabolism, including infectious, inflammatory, malignant, and ischemic conditions. Uptake values are typically higher in patients with STB compared with those with pyogenic spondylitis, [15] and small studies suggest that PET/CT may be superior to MRI in accurately diagnosing STB [16].

One of the objectives of the Spinal TB X cohort is to characterize the clinical and radiographic phenotype of STB—differentiating isolated from disseminated forms—using whole-body PET/CT imaging. [17] In this report, we describe the demographics, clinical presentation, PET/CT findings, and microbiological results from the first ten microbiologically confirmed STB cases enrolled in the cohort.

To date, no prospective cohort studies have systematically applied whole-body PET/CT to STB, and the clinical and radiographic spectrum of isolated versus disseminated disease remains poorly defined.

## Material and methods

### Study design, setting, and eligibility

The Spinal TB X cohort (NCT05610098) is a prospective study of HIV-infected and HIV-uninfected patients with STB residing in the Cape Town metropole, South Africa. Patients with suspected STB were referred from Groote Schuur Hospital and its affiliated hospitals (Mitchell’s Plain Hospital, New Somerset Hospital, and Victoria Hospital Wynberg) to the study team at the University of Cape Town.

### Patient recruitment

Patients were eligible if they had clinical and MRI features suggestive of STB and fulfilled the cohort inclusion criteria, as described in the published study protocol [17]. Demographic and clinical data, HIV status, and history of previous TB episodes were recorded. Microbiological and histological investigations were performed on accessible sites of disease, including sputum, urine, spinal specimens, and other sites where biopsy could be obtained. These specimens were tested using GeneXpert Ultra and MGIT culture. Haematological parameters including C-reactive protein (CRP), CD4 cell count (for HIV-infected patients), and full blood count were also recorded.

### Case definition

Isolated STB was defined as spinal osteomyelitis, including associated (e.g., psoas) abscess, caused by *Mtb* without evidence of lesions elsewhere in the body. Disseminated STB was defined as spinal disease with additional organ involvement due to *Mtb.*

### PET/CT acquisition and imaging analysis

Whole-body PET/CT was performed at the Cape Universities Body Imaging Centre, University of Cape Town, using a Siemens Biograph mCT 64 flow PET/CT system. Scans were acquired with the following parameters: 120 kV, 200 mA, 0.75 s rotation time, pitch 0.438, and collimation 16 × 0.75 mm. The CT scan was done without contrast. After fasting for 6 h and confirmation of normal blood glucose, patients received an intravenous injection of 2.8 MBq/kg body weight of fluorodeoxyglucose (FDG). After approximately 50 min, patients voided urine, and whole-body PET/CT was performed 60 min after injection.

Images were analysed using MIM^*®*^ software (Version 7.2.8; MIM Software Inc., Cleveland, Ohio, USA). Lesions were assessed by spinal level (cervical [C1–7], thoracic [T1–12], lumbar [L1–5], and sacral [S1–3]), presence of psoas abscess, number of lesions per spinal column, and number of affected vertebrae per lesion. For each lesion, SUVmax, SUVmean, and total lesion glycolysis (TLG) were recorded.

**Fig. 1:**
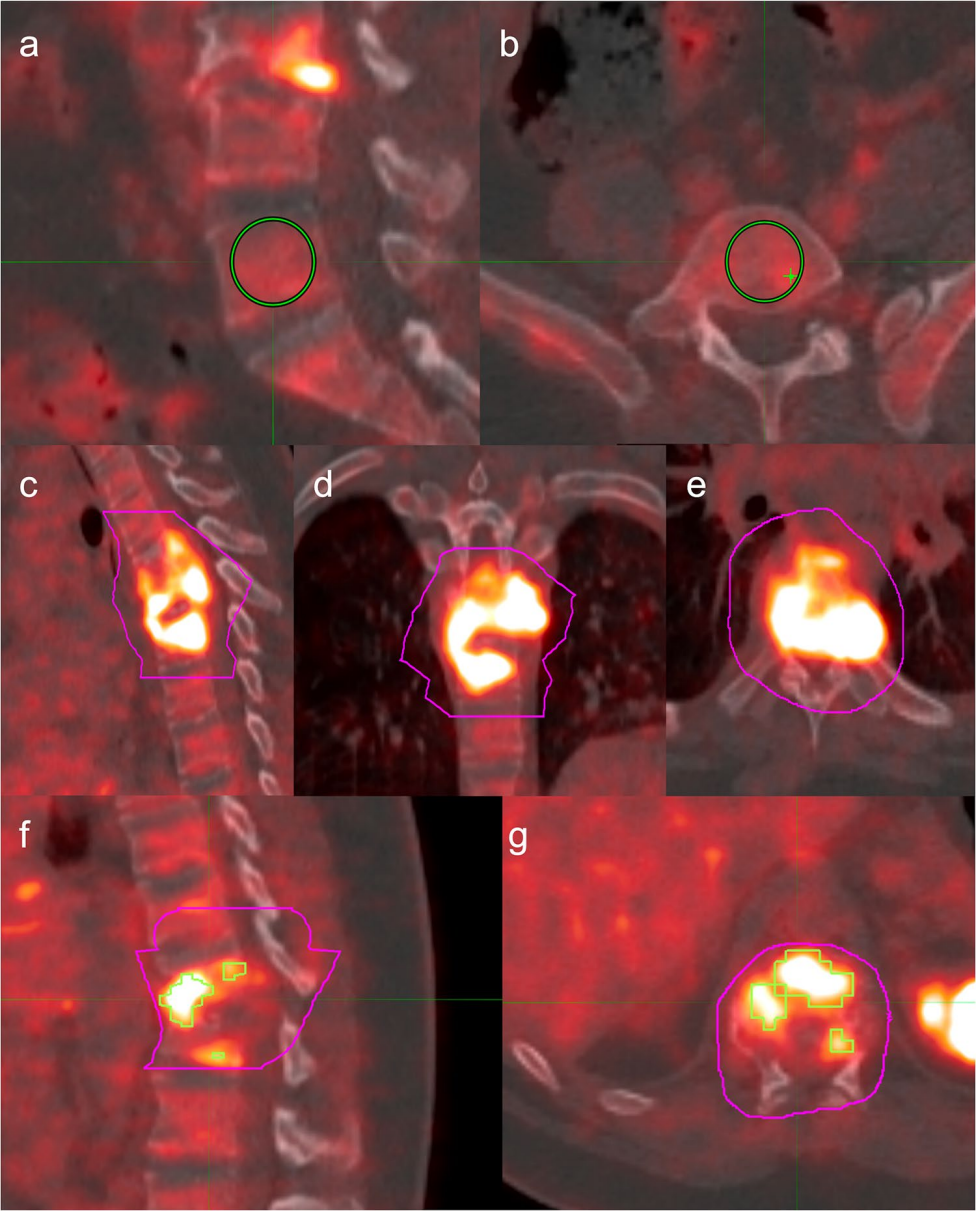
*PET/CT reading algorithm*. **a**, **b**: A 2D-ROI (Region of Interest) is placed in the central aspect of every not apparently diseased vertebra on sagittal (3A) and axial plane (3B) with maximum radius including only cancellous bone. **c**–**e**: After determination of the subthreshold value, an ROI is drawn using a 2-dimensional or a 3-dimensional brush around each lesion (3C sagittal plane; 3D coronal plane; 3E axial plane). **f**, **g**: New ROI (green) for SUVmax, SUVmean and TLG measurement after applying the patient-specific subthreshold (3F sagittal plane; 3G: axial plane)

To adjust for systemic FDG uptake due to TB-associated inflammation and bone marrow activation, [18, 19] all apparently unaffected vertebrae were assessed by placing a spherical region of interest (ROI) in the central cancellous bone (Fig. 1A, B). Mean SUVmax across these vertebrae plus two standard deviations (SD) was used as a patient-specific subthreshold, reflecting uptake not attributable to local *Mtb* infection. ROIs were then applied to each lesion (Fig. 1C–G) using this subthreshold to calculate SUVmax, SUVmean, and TLG directly attributable to local infection. This method minimized bias due to systemic FDG distribution, ensuring comparable measurement of spinal lesions even in patients with high extraspinal uptake, for example, in the presence of large pulmonary lesions with extensive FDG avidity, where systemic FDG “loss” would otherwise occur.

### Statistical analysis

All analyses were performed using SPSS Statistics for Mac, version 26.0 (IBM, Chicago, IL, USA). Data are presented as frequencies (n), means with standard deviation (SD), or medians with interquartile range (IQR). Group comparisons were made using χ^2^ tests for categorical variables, Student’s *t* tests for normally distributed continuous variables, and Mann–Whitney U tests for non-normally distributed variables. A two-sided *p* value < 0·05 was considered statistically significant.

### Ethical considerations

The study was approved by the Human Research Ethics Committee, Faculty of Health Sciences, University of Cape Town (HREC 243/2022). Written informed consent was obtained from all participants.

## Results

The cohort comprised the first ten patients enrolled in the Spinal TB X study (Table 1) . All were of Sub-Saharan African ancestry with a median age of 44 years (IQR 26, range 27–64 years) and male predominance (80%). Three patients (30%) were HIV-positive, with CD4 counts ranging from 320 to 838 cells/µL, and two of them had a history of previous pulmonary TB.

**Table 1:** Clinical, microbiological, and histological characteristics of patients with microbiologically confirmed spinal tuberculosis in the Spinal TB X cohort

Patient ID	Sex	Age	HIV-status	CD4 count (cells/µL)	GeneXpert ultra result spinal specimen	CT-value	MGIT culture result spinal specimen	Time to culture positivity in days	Drug resistance	Histological findings	Comment
TBX0001	M	27	Negative	N/A	Positive	28	Negative	N/A	not detected	Focal granulomatous inflammation	
TBX0002	M	64	Positive	838	Positive	21	Negative	N/A	not detected	Coalescing granulomas, central necrosis, multinucleated giant cells	Previous TB
TBX0007	M	48	Positive	320	Positive	20	Positive	27	not detected	Necrotising granulomatous inflammation	Previous TB
TBX0009	F	40	Negative	N/A	Positive	22	Positive	28	not detected	Necrotizing granulomatous inflammation	
TBX0010	F	49	Negative	N/A	Positive	23	Negative	N/A	not detected	Giant cells, vague granuloma formation, extensive suppurative inflammation	Salmonella co-infection
TBX0011	M	25	Negative	N/A	Positive	20	Positive	20	not detected	N/A: aspirate only	Psoas abscess
TBX0012	M	56	Negative	N/A	Positive	18	Positive	29	not detected	Focal necrosis	
TBX0013	M	62	Negative	N/A	Positive	16	Positive	4	not detected	Necrotizing granulomatous inflammation, multinucleated giant cells, Ziehl–Neelsen positive for AFBs	
TBX0014	F	33	Positive	487	Positive	18	Positive	21	*Rifampicin, isoniazid	N/A: aspirate only	Psoas abscess
TBX0016	M	33	Negative	N/A	Positive	18	Positive	6	not detected	N/A: aspirate only	Psoas abscess

CT-value, cycle threshold value of GeneXpert Ultra; MGIT, Mycobacteria Growth Indicator Tube; AFB, Acid-Fast Bacilli; *MDR-TB, Rifampicin resistance on GeneXpert Ultra, and Rifampicin plus Isoniazid resistance on MGIT culture using Hain MTBDRplus

### PET/CT imaging findings

**Fig. 2:**
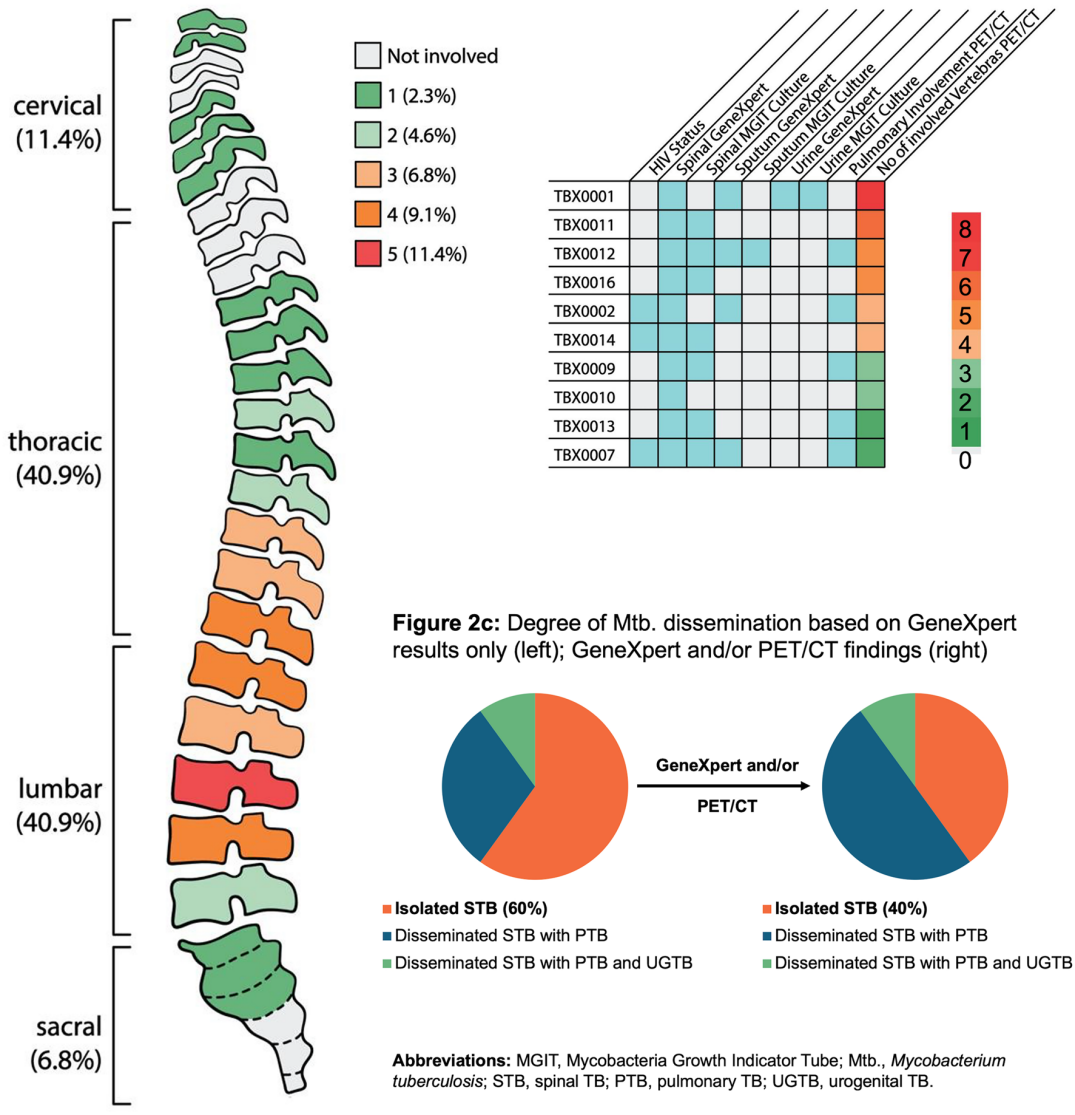
Degree of dissemination in ten patients with spinal tuberculosis (TB)—isolated spinal TB versus disseminated spinal TB using 18F-FDG PET/CT, Gene Xpert Ultra and MGIT culture results

On PET/CT, the thoracic and lumbar spine were the most frequently affected regions. The anatomical distribution of diseased vertebrae is shown in Fig. 2a. Seven patients had a single spinal lesion, two patients had two lesions, and one patient had three lesions, resulting in a total of 14 lesions across the cohort (Table 2). The mean lesion count per spinal column was 1.4 (SD 0.7). The cumulated number of diseased vertebrae was 44. The mean number of diseased vertebrae per patient was 4.4 (SD 1.7), and the mean number of diseased vertebrae per lesion was 3.1 (SD 1.4). Psoas abscesses were present in six patients.

**Table 2:** PET/CT characteristics of spinal lesions in patients with microbiologically confirmed spinal tuberculosis

Patient ID	Number of infected vertebras (n = 44)	Number of lesions (n = 14)	Lesion 1	TLG	SUVmax	SUVmean	Lesion 2	TLG	SUVmax	SUVmean	Lesion 3	TLG	SUVmax	SUVmean
TBX0001	8	3	T4–7	397.4	17.4	9.4	T11–12	137.7	17.6	7.9	L3–4	23.5	11.8	7.5
TBX0002	4	1	T7–10	834.2	18.5	7.4								
TBX0007	2	1	L3–4	61.0	6.2	3.3								
TBX0009	4	1	T10–L1	241.8	15.2	7.1								
TBX0010	3	1	C5–7	190.2	12.8	6.5								
TBX0011	6	1	T9–L2	386.9	19.0	6.4								
TBX0012	5	2	C1–2	8.9	6.6	4.9	L1–3	1030.9	18.4	7.9				
TBX0013	3	2	T11–12	591.0	19.7	7.0	L3	37.1	12.9	6.7				
TBX0014	4	1	L2–5	273.8	15.7	5.9								
TBX0016	5	1	L4–S3	870.0	21.2	8.7								

**Fig. 3:**
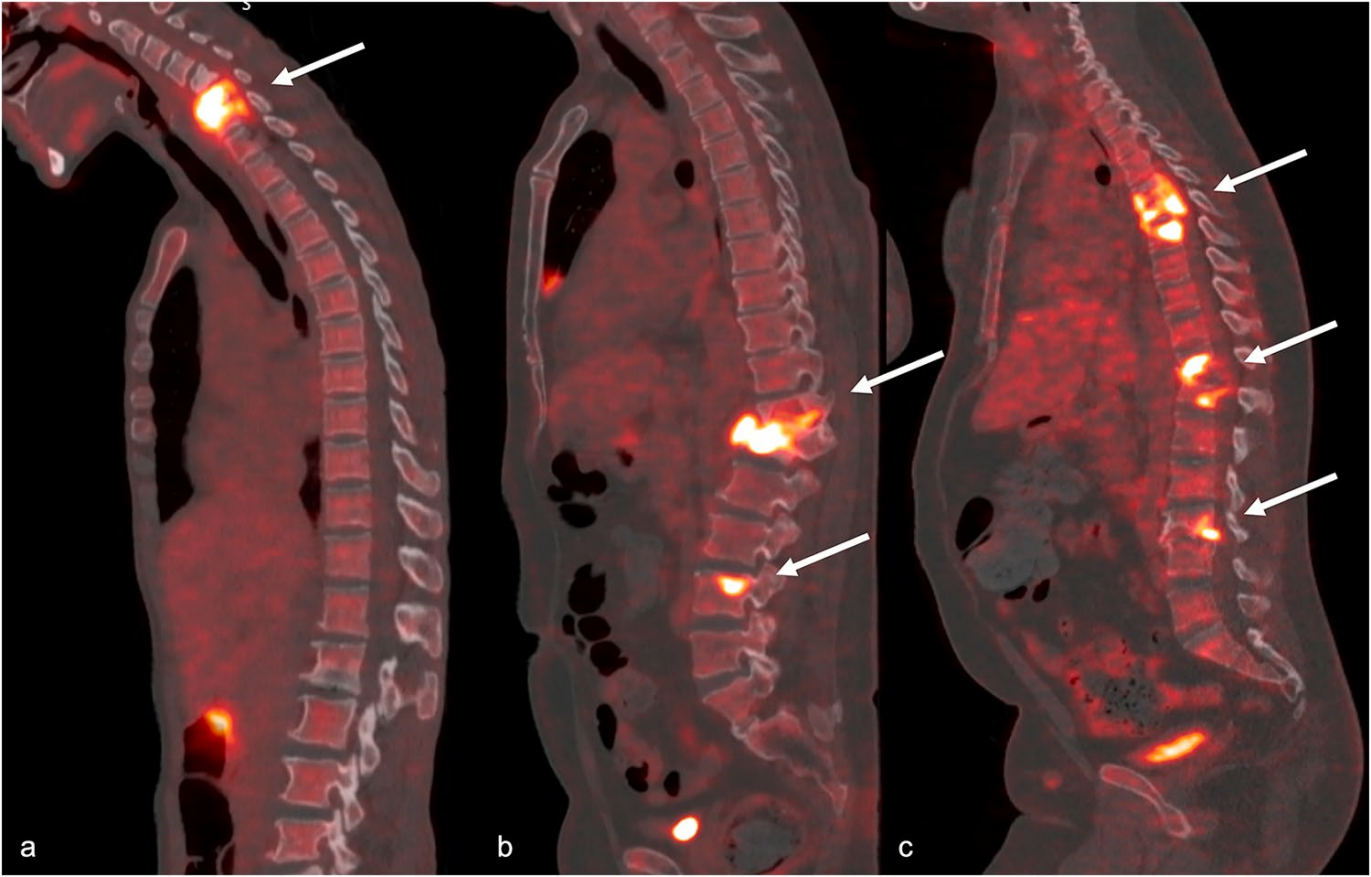
*PET/CT-imaging of three patients with different amounts of spinal lesions*. **a** Patient with three non-contiguous spinal lesions, **b** Patient with two non-contiguous spinal lesions, **c** Patient with singular spinal lesion

Across all 14 spinal lesions, the mean total lesion glycolysis (TLG) was 363.2 SUVbw*ml (SD 341.9, range 8.9–1030.9). The mean SUVmax was 15.2 (SD 4.6, range 6.2–21.2) and the mean SUVmean was 6.9 (SD 1.5, range 3.3–9.4). Figure 3 illustrates examples of a patient with a single spinal lesion (A), a patient with two non-contiguous lesions (B), and a patient with three non-contiguous lesions (C).

### Microbiological and histological findings of spine samples

Spinal samples were collected in all 10 patients for the definite diagnosis of spinal TB. All spinal biopsy samples (aspirates n = 3, in case of easier access to psoas abscess) were GeneXpert Ultra positive with CT values ranging from 16.4 to 27.5 (mean CT value 20.3, SD 3.2). Three out of ten patient’s spinal lesion biopsy specimens were negative on MGIT culture growth. In the seven patients with positive MGIT culture results, the time to positivity (TTP) ranged from 4 to 29 days (mean 19.3 days, SD 10.4). All GeneXpert Ultra results were sensitive for rifampicin and isoniazid except for TBX0014, who had multi-drug-resistant TB (rifampicin resistance on GeneXpert Ultra; rifampicin and isoniazid resistance on MGIT culture using Hain MTBDRplus). TBX0010 was diagnosed with bacterial co-infected spondylodiscitis due to *Salmonella* (non-Typhi) identified in the spinal biopsy specimen. Histology was available in all patients who underwent spinal biopsy (n = 7). Four of the seven biopsy specimen showed histopathological evidence of TB infection (Table 2).

### Degree of dissemination in Spinal TB according to microbiological results

All ten patients were GeneXpert Ultra positive on spinal specimens, and seven also had a positive spinal MGIT culture (Fig. 2b). Four patients had additional positive GeneXpert Ultra results from sputum, including one who was also MGIT culture positive. Among these, one patient further tested positive in urine by both GeneXpert Ultra and MGIT culture. Based on GeneXpert Ultra results from spinal, sputum, and urine specimens, six patients (60%) had isolated spinal TB (STB), three had STB with concomitant pulmonary TB, and one had STB with both pulmonary and urogenital TB (Fig. 2b, c).

### Degree of dissemination of Spinal TB according to microbiological results and PET/CT findings

**Fig. 4:**
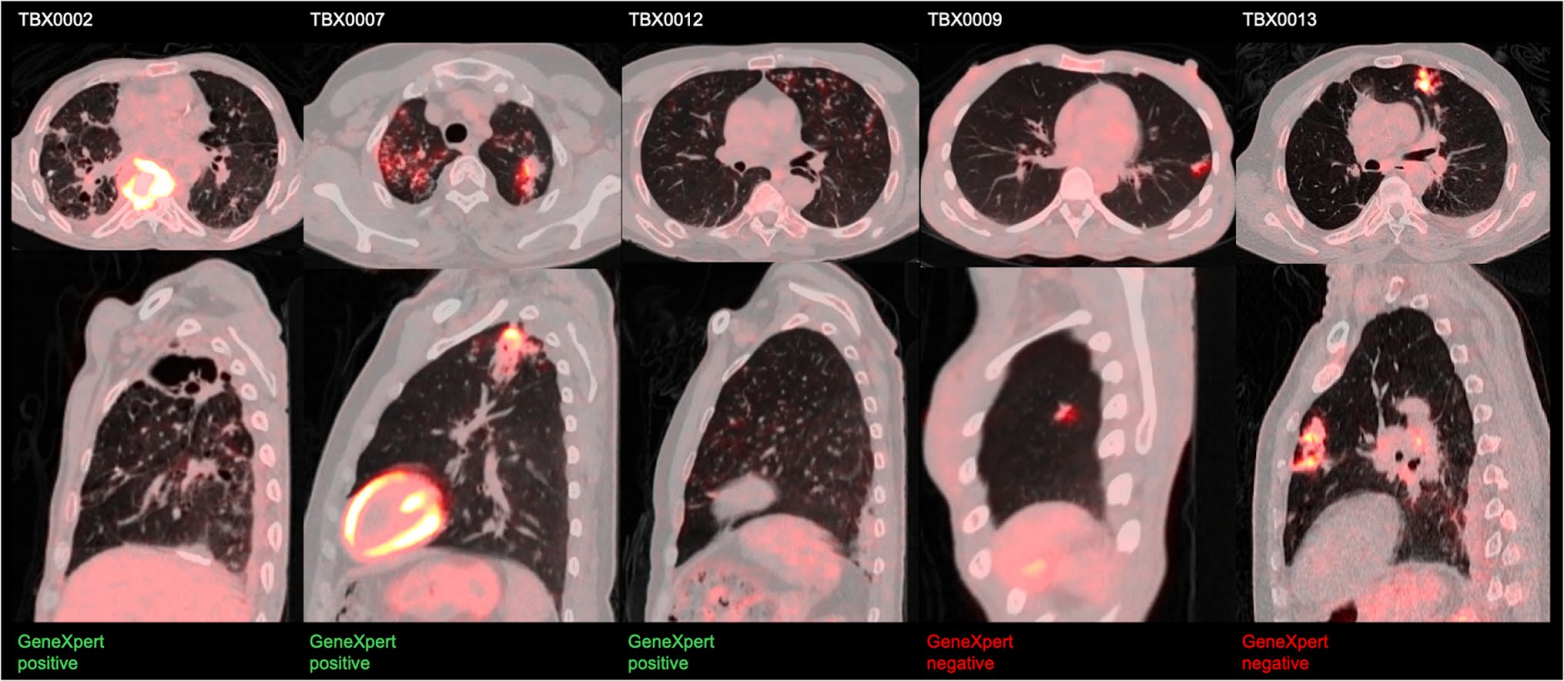
PET/CT imaging of the five patients with increased pulmonary FDG-uptake

Five patients demonstrated increased pulmonary FDG uptake on PET/CT, of whom three also had a positive sputum GeneXpert result (Fig. 4). Figure 2b summarizes HIV status, overall microbiological results, pulmonary involvement on PET/CT, and the extent of spinal disease by number of affected vertebrae. When combining microbiological and PET/CT findings, 40% of patients had isolated STB, whereas 60% had disseminated disease.

## Discussion

In this prospective cohort study, we systematically applied whole-body FDG-PET/CT to patients with microbiologically confirmed STB. We found that disseminated disease was common, affecting 60% of patients when microbiological results were combined with PET/CT findings. This underscores the systemic nature of tuberculosis, even when clinical presentation appears to be confined to the spine. To our knowledge, this is the first study to characterize the clinical and radiographic phenotype of spinal TB using whole-body PET/CT, thereby providing novel insights into disease extent and potential applications for diagnosis and treatment monitoring.

HIV infection is one of the strongest risk factors for developing disseminated TB. [20, 21] Reported rates of HIV co-infection among patients with STB vary widely, ranging from 27 to 76% across different settings [22–26]. In our case series, 30% of patients were HIV-positive, a proportion consistent with the lower end of this range. Two of the three HIV-infected patients had reduced CD4 cell counts, although all remained above the AIDS-defining threshold of < 200 cells/µL. [27] Notably, two of these patients had a prior history of pulmonary TB, in line with the well-established association between immunosuppression and recurrent TB disease. However, this proportion was lower than reported in previous studies, which found that more than half of patients with STB had either concurrent or past pulmonary TB [10, 28].

The key findings of this case series are as follows. First, based on microbiology alone, 40% of patients had disseminated STB. Second, when combining microbiological and PET/CT findings, 60% had disseminated disease. This is consistent with the pathophysiology of STB, which typically arises from hematogenous spread of *Mtb* from a primary pulmonary focus. [29] Our results therefore support the recommendation that sputum testing should be performed in all patients with suspected STB, even in the absence of clinical symptoms of pulmonary TB.

Previous studies have reported concomitant active pulmonary TB in 2 to 18% of patients with STB when defined by sputum culture. [30–34] In our cohort, the incidence was nearly three-fold higher, although this was based on GeneXpert Ultra results and FDG-avid pulmonary lesions on PET/CT rather than culture positivity alone. GeneXpert Ultra positivity in conjunction with FDG-avid pulmonary lesions without culture confirmation may reflect healed or contained lesions with residual metabolic activity, ongoing subclinical replication, or paucibacillary disease that escapes detection[35–37].

Interestingly, several patients had isolated STB without radiological or microbiological evidence of pulmonary involvement. This raises the possibility that isolated STB represents a distinct clinical phenotype or even a separate disease entity. Potential explanations include infection with specific *Mtb* lineages with reduced pulmonary tropism and enhanced adaptation to hypoxic conditions [38, 39] differential expression of virulence factors enabling survival in spinal tissue, [40, 41] or host genetic factors that predispose to extrapulmonary dissemination without lung involvement. [41, 42] In future research, we aim to integrate pathogen genomics and host transcriptomics to clarify these mechanisms [17].

Few studies have examined PET/CT findings in STB. Four previous reports described mean SUVmax values ranging from 10.2 to 17.8, which align with our observed mean SUVmax of 15.2. [43–50] However, cross-study comparisons must be interpreted with caution because variability in scanners, protocols, and patient biology can significantly affect uptake measurements. [51] Of note, two of our patients with confirmed pulmonary TB and increased pulmonary FDG uptake had markedly lower SUVmax values in their spinal lesions, possibly reflecting systemic competition for FDG in the presence of multiple metabolically active sites. Importantly, to our knowledge, this is the first study to report total lesion glycolysis (TLG) in bacterial infections of the spine. In oncology, TLG, a volume-based parameter, has demonstrated superior prognostic value compared with single-voxel measures such as SUVmax, as it better reflects the overall metabolic burden of disease. [52–54] Emerging evidence suggests similar prognostic utility of TLG in infectious diseases. [55] Our findings suggest that TLG warrants further evaluation as a quantitative biomarker for disease monitoring and treatment response in STB. Within this cohort, patients will undergo repeat PET/CT imaging at 6 and 12 months of antimicrobial therapy to assess metabolic changes over time.

### Limitations

The small sample size limited statistical power and subgroup analyses. Only microbiologically confirmed STB cases were included, excluding clinically diagnosed STB. Culture yields varied by biopsy technique and operator experience, which could not be standardised. The single-centre setting in Cape Town and referral from tertiary hospitals may limit generalisability and introduce selection bias. PET/CT is resource-intensive and not widely available in high TB-burden regions, restricting clinical applicability. The absence of comparator groups with pyogenic or malignant lesions limits the assessment of diagnostic specificity.

## Conclusion

In this first prospective application of whole-body PET/CT to patients with microbiologically confirmed STB, we found that a substantial proportion had disseminated disease, while others exhibited isolated spinal involvement without pulmonary manifestations. These findings suggest that isolated STB may represent a distinct disease phenotype. PET/CT offers unique value as a whole-body imaging modality for assessing disease dissemination and quantifying metabolic burden. Based on our findings, sputum testing should be performed in all patients with suspected STB. Future studies should investigate the pathogen and host-specific factors underlying disseminated versus isolated STB and evaluate the prognostic role of PET/CT parameters, particularly TLG, in larger, prospective cohorts using standardized imaging and sampling protocols.

## Data Availability

Data is available on reasonable request.
